# The Alleviative Effect of Oral Administration of *Lactiplantibacillus plantarum* 22A-3 on Allergic Contact Dermatitis in Mice

**DOI:** 10.3390/nu18203446

**Published:** 2026-10-20

**Authors:** Ken-ichiro Minato, Miho Chikazawa, Kyoka Unno, Yoshie Yamana, Takashi Tagawa, Hiroshige Kuwahara, Masashi Mizuno

**Affiliations:** 1 Department of Applied Biological Chemistry, Graduate School of Agriculture, Meijo University, Nagoya 468-8502, Japan; mchika@meijo-u.ac.jp; 2 Department of Applied Biological Chemistry, Faculty of Agriculture, Meijo University, Nagoya 468-8502, Japan; 3 Research Center, Maruzen Pharmaceuticals Co., Ltd., Hiroshima 729-3102, Japan; y-yamana@maruzenpcy.co.jp (Y.Y.); t-tagawa@maruzenpcy.co.jp (T.T.); h-kuwahara@maruzenpcy.co.jp (H.K.); 4 Department of Health and Nutrition, Faculty of Human Science, Osaka Aoyama University, Minoh 562-8580, Japan; mizuno@kobe-u.ac.jp

**Keywords:** allergic contact dermatitis, anti-inflammation, KikGR mice, *Lactiplantibacillus plantarum* 22A-3, macrophage

## Abstract

Background/Introduction: The oral administration of *Lactiplantibacillus plantarum* 22A-3 (LP22A-3) could suppress ear inflammatory edema in the mice sensitized with an allergy-induced chemical compound, 1-fluoro-2, 4-dinitrobenzene (DNFB). Method: In this study, we formulated a hypothesis that the homeostasis of immunocompetent cells might be associated with alleviating this inflammation. Results: Pro-/anti-inflammatory cytokines are involved in the cell differentiation in vitro. Our results showed that the accumulation of pro-inflammatory cytokines, TNF-α and IL-6 were attenuated by the administration of the LP22A-3, in which bacteria were inactivated by heating at 80 °C. On the other hand, the level of the anti-inflammatory one, IL-10, was maintained. These findings suggested that LP22A-3 could be induced to build anti-inflammatory conditions. Regarding the cell migration from the small intestine, we aimed to clarify the mechanism in more detail by conducting experiments using KikGR mice to track the migration of target cells. We found that the migration of CD11b^+^CD86^+^ cells from the ileum increased in the DNFB-induced allergic dermatitis in mice. Then, the oral LP22A-3 administration could reduce their levels in the ileum to baseline levels. On the other hand, the migration of CD11b^+^CD206^+^ cells was enhanced in the dermatitis mice. However, it was shown that the increase in migration of those cells was not influenced by the LP22A-3 administration. Furthermore, our results suggested that oral LP22A-3 administration inhibited the differentiation of CD4^+^ cells into IL-9-inducing cells. Conclusions: This study might show the potential of immunocompetent cell action for alleviating the inflammation. In addition, the oral LP22A-3 administration could be a promising alternative alleviative strategy for type-IV allergic disorders.

## 1. Introduction

Contact dermatitis (CD) is a common cutaneous inflammatory disorder resulting from stimulation with exogeneous substances. The major risk factors for CD are environmental factors such as contact of allergens and exposure of irritants stemming from household activities, social, and skin maceration [1]. Allergic contact dermatitis (ACD) is one of the main forms of CD and known as a Type IV allergy triggered by delayed-type hypersensitive reaction [2,3]. Allergens such as haptens, which are low-molecular-weight chemicals, penetrate the epidermis, and bind to proteins in the skin. Scientific data shows that nearly 3000 chemical substances in the environment can trigger ACD. Antimicrobial ingredients, preservatives, and surfactants are included in the contact allergens [4]. When these antigenic-inducing substances encounter the skin in the broad context of the environment, their immunogenicities are exerted as sensitizers. The hapten-protein complexes stimulate the immune system and provoke sensitization in ACD. During the induction phase (sensitization phase), the skin is contacted with a hapten, which then stimulates Langerhans cell (LC) movement from the epidermis via the afferent lymphatic vessels to the skin-draining lymph nodes [3]. Haptenized LCs come into the paracortical area where the T cells are rich. These T cells specifically recognize allergen-MHC molecule complexes and then expand abundantly and generate effector and memory T cells [3,5]. They are released via the efferent lymphatics into the circulation system. The effector T cells express acquired homing receptors and can be recruited locally at the site of secondary hapten challenge (elicitation phase). The elicitation phase is also called the efferent phase. During this phase, the hapten contacts the same area of skin after the sensitization phase finishes. In addition, the hapten-specific T cells are triggered to secrete pro-inflammatory cytokines and chemokines by action of various haptenized cells including LC and keratinocytes (KC). Therefore, more inflammatory cells are recruited and further release of inflammatory mediators are amplified locally. The common and prominent clinical symptom of ACD is pruritus. Morphologically, the lesion of ACD shows erythema and edema at first (acute stage), possibly within 24–72 h, then crust and scales (subacute stage), and ultimately lichenification (chronic stage) [3,6,7]. Inflammation is also a significant symptom in ACD pathogenesis. It is a host defense mechanism and a complex immune response that are mediated by inflammatory immune cells to protect the body from invading pathogens. It often shows several hallmark signs, including redness, swelling, heat, pain, and organ dysfunction. Several studies have reported that inflammatory responses during allergic reaction in skin are initiated through the breakdown in the balance between T cells responses, Th1/2 balance [5,8,9,10].

Several tissues such as dermis and intestine contain matured monocyte-derived macrophages [11]. These are tissue-resident macrophages, which exhibit highly heterogeneous characteristics by adapting to the local environments of their respective organs and tissues. Upon stimulation by various cytokines and growth factors, these cells can differentiate from peripheral blood monocytes. Furthermore, during infection, monocytes activated in the bloodstream migrate into inflamed tissues and differentiate into specific monocyte-derived macrophage populations that contribute to the restoration of tissue integrity and the elimination of pathogens [12]. Macrophages can be classified into two types: M1 macrophages (classically activated macrophages) and M2 macrophages (alternatively activated macrophages) [3,13,14]. M1 macrophages express Toll-like receptor (TLR)-4 and produce tumor necrosis factor (TNF)-α, interleukin (IL)-6, IL-1β, and IL-12 via the NFκB pathway in response to IFNγ. Four distinct subcategories comprise the M2 macrophages population. They are M2a, M2b, M2c, and M2d. M2 macrophages are characterized by the release of arginase-1 (Arg1) in mice and by the suppression of inflammatory cytokines [13]. M1 macrophages could lead to pathogenesis of ACD in response to an action of IFNγ. In contrast, through a Th2-skewed mechanism, M2 macrophages have the potential to suppress ACD. It has been reported that skin biopsies obtained from ACD patients express iNOS^+^CD14^+^ M1 macrophages, and that iNOS contributes to ACD-associated inflammation [15]. Furthermore, mice lacking Arg1 in macrophages show exacerbated ear thickness and dermal inflammation. Previously, it has been reported that CX3CR1-deficient mice show attenuated ACD-associated responses, as measured by ear thickness in responses to 1-fluoro-2, 4-dinitrobenzene (DNFB), which is a typical inducer of delayed-type allergic dermatitis, with decreased expression levels of M1 macrophage markers such as TNF-α, IL-6, and iNOS. Moreover, expression levels of M2b macrophages markers including Arg1 increased in the ACD mice. Those results have suggested that the skewed polarization towards M2 phenotype leads to attenuate the contact hypersensitivity response [16]. Further studies on each M2 macrophage subtype may help clarify their specific roles in alleviating ACD.

Developing efficacy-driven therapeutics for ACD remains a primary focus of current research. It has been shown that food factors can affect the development and alleviation of the contact dermatitis [2,17]. A lot of probiotic strains, whose effective health impacts have been well-known, are isolated from traditional fermented foods [18,19]. Although food-derived bacteria such as Lactobacillus and Bifidobacterium species are frequently used as probiotics, different strains display distinct activities. Therefore, the use of probiotics requires a greater understanding of their specific activities in alleviating allergic responses. In the previous study, the oral administration of pickle-derived *Lactiplantibacillus plantarum* 22A-3 (LP22A-3) suppressed the development of dermatitis induced by 2,4-dinitrochlorobenzene which was used to develop atopic dermatitis-like lesions in NC/Nga mice [20]. Moreover, in co-culture system composed of Caco-2 and RBL-2H3 cells, LP22A-3 administration on Caco-2 cells before or after the sensitization to an allergen indicated the different effect on inflammatory factors release from RBL-2H3 [21]. This co-culture system is a promising in vitro assessment model reflecting the intestinal environment, as an alternative to animal experiments. LP22A-3 administration before the sensitization decreased a pro-inflammatory factor release, but not after sensitization, indicating that LP22A-3 affected effector cells (mast cells) sensitized with allergen through intestinal epithelial cells. These results suggest that LP22A-3 possesses a prospective alleviative property for an allergic response.

In this study, we explored the pharmacological effect of LP22A-3 on the pathogenesis of ACD caused by DNFB sensitization. We here indicate that the oral administration of LP22A-3 can suppress edema formation through the modulation of macrophage differentiation. Moreover, we utilized KikGR knock-in mice (expressing a photoconvertible protein) [22,23] to trace macrophage migration from the small intestine to the proximal area of inflammatory site. The current study indicates that the inhibitory effect of oral intake of LP22A-3 on ACD may be mediated by macrophages crosstalk between the small intestine and the inflammatory site.

## 2. Materials and Methods

### 2.1. Lactiplantibacillus Plantarum 22A-3 (LP22A-3)

LP22A-3 was cultured in Mann–Rogosa–Sharpe broth and incubated overnight at 30 °C in an anaerobic chamber. After incubation, bacterial cells were collected by centrifugation (4 °C, 10,000× *g*, 5 min) and were resuspended in PBS. Then, to eliminate residual culture medium, they were washed twice. The bacterial suspension was inactivated by heating at 80 °C for 30 min due to preventing contamination of the culture system by secretions from viable LP22A-3. The inactivated bacteria were lyophilized and kept at −20 °C until investigation.

### 2.2. Cell Culture

Caco-2, which is known as a human intestinal epithelial cell line, provided by RIKEN BRC (Tsukuba, Japan), was cultured in DMEM (glutamine, high glucose), supplemented with 1% MEM-NEAA, 100 U/mL penicillin, 100 μg/mL streptomycin, and 10% decomplemented FBS (56 °C, 30 min). The human macrophage cell line, THP-1, was cultured in RPMI1640 supplemented with 100 U/mL penicillin, 100 μg/mL streptomycin, 2 mM glutamine, and 10% FBS. The THP-1 cells, provided by RIKEN BRC (Tsukuba, Japan), were differentiated to naïve macrophages according to the protocol by Chanput et al. [24], as follows. Their macrophage-like states were obtained by treating the monocytes (1 × 10^6^ cells) for 48 h with 160 nM of phorbol 12-myristate 13-acetate (PMA) in 24-well cell culture plates. Then, differentiated and adherent cells were washed twice with RPMI1640 medium containing 10% FBS. To obtain the resting state of macrophage, they were incubated for another 24 h in RPMI1640 medium excluding FBS. Subsequently, the cultures were washed to remove the nonadherent cells.

### 2.3. Co-Culture System

To evaluate anti-inflammatory effect of LP22A-3, an in vitro assay using a Caco-2/THP-1 coculture system, which had been already established by our group [25], was performed. Caco-2 cells were seeded at 9 × 10^4^ cells/cm^2^ onto a 12-well Transwell insert plate (0.4 μm pore size). The cell culture medium was changed every 3 days until the trans-epithelial electrical resistance (TEER) value was >250 Ω·cm^2^, and the cells were used in passage numbers 48–62. The differentiated naïve THP-1 cells were seeded at 8 × 10^5^ cells/cm^2^ in 12-well tissue culture plates and incubated overnight to ensure complete adherence to the well, before being used in passage numbers 10–37. After replacing the medium for THP-1 cells with RPMI1640 and the medium for Caco-2 cells with RPMI1640 in the presence or absence of LP22A-3, the Transwell inserts were added to multiple plate wells preloaded with the THP-1 cells. Following a 3 h incubation period, lipopolysaccharide (LPS) was applied to the basolateral chamber of this co-culture model. After additional incubation, culture supernatants from the basolateral side were collected and kept at −80 °C until measurement of the cytokine production.

### 2.4. Determination of Cytokines

The secretion of pro- and anti-inflammatory mediators in the co-culture system was determined by ELISA. TNF-α, IL-6 and IL-10 levels were determined by using anti TNF-α, IL-6 and IL-10 antibodies as previously described [26]. Briefly, a maxisorb 96-well plate was coated with coating Ab of each cytokine overnight at 4 °C. After a 30 min blocking step at 37 °C, the culture supernatant and detection antibody were introduced and incubated for 2 h at room temperature (RT) with orbital shaking at 500 rpm. Simultaneously, cytokine concentration standards were tested. Following four washes with PBS, the plate was incubated with Streptavidin–HRP (ThermoFisher Scientific, MA, USA) for 30 min. The reaction was developed by Tetramethyl Benzidine (TMB, Dojindo, Kumamoto, Japan) substrate and optical density measured by a spectrophotometer (450 nm).

### 2.5. Mice

This study was approved by the Institutional Animal Care and Use Committee of Meijo University (Permission number: 2025PE3, approval date: 31 March 2025) and carried out in accordance with the Meijo University Animal Experimentation Regulations. Male 6-week-old C57BL/6NCrSlc mice were purchased from Japan SLC (Shizuoka, Japan). Prior to each study, the mice were randomly group-housed in polycarbonate cages (*n* = 5 per group), and were then habituated for one week, during which a basal diet (DC-8; Clea Japan, Tokyo, Japan) and tap water were provided *ad libitum*. Animals were maintained at institutional animal facility of Meijo University in a climate-controlled room at 23 ± 2 °C and at 50 ± 10% humidity, under a 14 h light and 10 h dark regimen, and then used for animal experiments. No animals died or reached the predefined humane endpoint (HEP) criteria requiring euthanasia, vomiting blood, convulsions, and sudden loss of appetite and weight, during the experimental period. At the end of the study, the mice were sacrificed by cervical dislocation under the anesthetic of 1.5% isoflurane.

### 2.6. Dose of LP22A-3 and Induction of Allergic CD in the Mice

The C57BL/6NCrSlc mice were orally administered 10^8^ cfu (colony forming unit; 1.31 × 10^12^ cfu/g) of LP22A-3 in a single dose every day for 21 days, in accordance with our previous report [21]. A cfu refers to viable counts before heat treatment. The mice in the control group were continuously fed only the DC-8 diet. On Day 15 and 16, the mice were consecutively sensitized on the shaved abdominal sites with 50 μL of 0.5% DNFB (1-fluoro-2, 4-dinitrobenzene, Fuji Film Wako Pure Chemical Co., Ltd., Osaka, Japan) in acetone:olive oil (4:1). Six and seven days after the second sensitization (Day 21 and 22), mice were consecutively challenged by painting the outer surfaces of both sides of each pinna with 20 μL of 0.5% DNFB. Vehicle groups received acetone:olive oil only. Each pinna was measured with a thickness gauge. The net pinna thickness was calculated at 24 h after the last challenge. Symptomatology of contact hypersensitivity was analyzed as ear thickness difference between at immediately prior to the first challenge and 24 h after the second challenge. All procedures were performed under anesthesia to prevent stress for the animals (1.5% isoflurane mixture oxygen). Each of the five groups included five independent mice (25 animals total). At the end of the study, the mice were sacrificed by cervical dislocation under the anesthetic of 1.5% isoflurane.

### 2.7. Cell Preparation from Spleen, Peritoneal Cavity, and Lymph Nodes of the Treated Mice

The C57BL/6NCrSlc mice were put under anesthesia by 1.5% isoflurane induction, and the cells were collected by peritoneal lavage with 10 mL of serum-free RPMI 1640 medium on Day 23 (24 h after the last challenge). Then the mice were sacrificed by cervical dislocation under the anesthetic of 1.5% isoflurane induction, and single-cell suspensions were prepared by gently homogenizing the axillary lymph nodes with a frosted-glass homogenizer. We analyzed the profiles of CD11b^+^CD86^+^ and CD11b^+^CD206^+^ cells. In addition, the spleen was harvested aseptically. The single-cell suspensions were obtained by disrupting the spleens with dissecting forceps in DMEM. After centrifuging, cells were cleared of erythrocytes by resuspension in a hypotonic lysis buffer for 30 min on ice. The cells were washed once with fresh medium and filtered using a 40 μm filter to obtain a mononuclear cell suspension. We analyzed the profiles of the CD4^+^CD25^+^Foxp3^+^ cells.

### 2.8. Differentiation into CD4^+^ IL-9-Producing Cells

The C57BL/6NCrSlc mice (male, 7 weeks old, Japan SLC) were orally administered 10^8^ cfu of LP22A-3 in a single dose every day for 21 days. The mice in the control group were continuously fed only the DC-8 diet. Each of the three groups included five independent mice (15 animals total). On Day 22, the mice were sacrificed by cervical dislocation under the anesthetic of isoflurane, and then the naïve CD4^+^ T cells were isolated from the lymphocytes of the spleen in the mice by positive selection with magnetic cell sorting using anti-CD4 microbeads (MACS, Miltenyi Biotec, Bergisch Gladbach, Germany). The prepared naïve CD4^+^ T cells were stimulated with 3 μg/mL of anti-CD3 and 5 μg/mL of soluble anti-CD28 and then polarized into CD4^+^ IL-9-producing cells under the condition with 3 ng/mL of human transforming growth factor (hTGF)-β, 20 ng/mL of IL-4, and 20 μg/mL of anti-IFNγ, which were prepared according to the protocol by Schanz et al. [27]. Every cell was examined by a flow cytometry using FITC (for CD4)- and APC (for IL-9)-mAbs.

### 2.9. Photoconversion and Surgical Procedures

All KikGR mice (B6.Cg-Gt (ROSA)26Sor < tm1.1(CAG-kikGR)Kgwa > mice [22,23] (male, 10–12 weeks old, provided by RIKEN BRC, Tsukuba, Japan)) used in this study were bred in specific pathogen-free facilities at Meijo University. Each of the three groups included five independent mice (15 animals total). The mice were given a daily administration of LP22A-3 and sensitized by DNFB as described above (Section 2.6). Under 1.5% isoflurane anesthesia, ileal cells underwent photoconversion on Day 17. To prevent light exposure, non-photoconverted regions were wrapped in aluminum foil. After laparotomy, a targeted section of the intestine was pulled outside the body and exposed to violet light (405 nm, 54 mW/cm^2^) on both sides, the front and the back, for 2 min to trigger photoconversion [28,29]. Pre-warmed PBS was topically applied to the exposed region throughout light exposure to prevent tissue desiccation. Violet light permanently changes the protein KikGR from green to red [25]. Following photoconversion, the abdominal wall was temporarily approximated using bulldog forceps, then the incision was completely sutured using Michel clips. No animals died or reached the predefined humane endpoint (HEP) criteria requiring euthanasia, vomiting blood, convulsions, and sudden loss of appetite and weight, post-operatively. On Day 23, the mice were put under anesthesia by 1.5% isoflurane induction, and the cells were collected by peritoneal lavage with 10 mL of serum-free RPMI 1640 medium. At the end of the study, the mice were sacrificed by cervical dislocation under the anesthetic of 1.5% isoflurane. To remove cell debris and aggregates, the prepared cell suspensions were passed through a 40-μm cell strainer. Fluorochrome-conjugated antibodies used in this study were purchased from BioLegend (San Diego, CA, USA). Prior to flow cytometric analysis, cell suspensions were washed in Dulbecco’s PBS containing 2% FBS. Next, cells were incubated with Mouse BD Fc Block™ (BD Pharmingen, NJ, USA) to block Fc binding. KikGR-green and red signals were detected using 525/40 and 585/42 bandpass filters, respectively, by CytoFLEX Cell Analyzer (Beckman Coulter, Brea, CA, USA).

### 2.10. Antibodies and Flow Cytometry

Fluorochrome-conjugated antibodies were purchased from BioLegends (San Diego, CA, USA). The cells were stained with FITC (for CD4)- and PE (for CD25)-mAbs for 15 min at 4 °C. For intracellular staining, cells were fixed and permeabilized using Mouse Perm/Wash Buffer set (BD Pharmingen) and subsequently stained with APC-conjugated anti-Foxp3 or IL-9 antibodies. For macrophage analysis, cells were stained with APC-conjugated anti-CD11b, APC/Cy7-conjugsted anti-CD86, and PE/Cy7-conjugated anti-CD206 mAbs. Stained samples were acquired using CytoFLEX Cell Analyzer (Beckman Coulter). Flow cytometry data were analyzed using Flowjo software v11.

### 2.11. Statistical Analysis

Results are reported as the mean ± S.E.M or S.D. Statistical evaluations were conducted using SPSS 24 software (IBM Corp., Armonk, NY, USA). A one-way analysis of variance (ANOVA) followed by Tukey’s post hoc test was conducted, with the significance levels defined in each respective figure legend.

## 3. Results

### 3.1. Effect of LP22A-3 on the Secretion of Pro/Anti-Inflammatory Cytokines In Vitro

**Figure 1:**
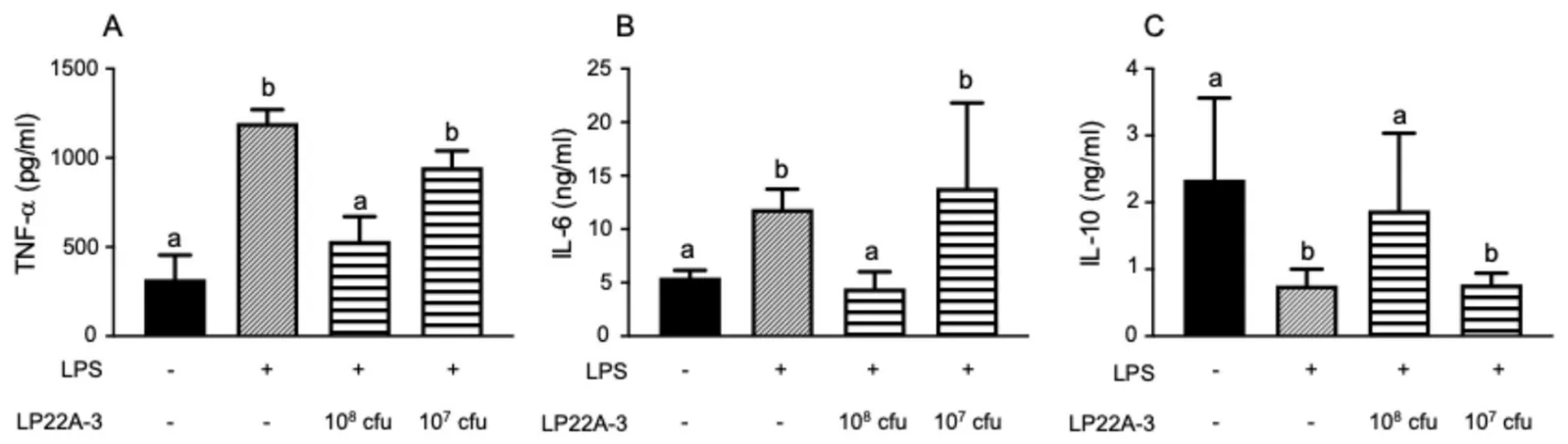
LP22A-3 attenuates TNF-α and IL-6, whereas enhances IL-10, in the Caco-2/THP-1 co-culture system. LP22A-3 (1 × 10^8^ or 1 × 10^7^ cfu/mL) was added into the apical compartment in the Caco-2/THP-1 co-culture system for 2 h. Subsequently, LPS was added to the basolateral compartment at a final concentration of 10 ng/mL and incubated for an additional 24 h. (**A**) TNF-α production, (**B**) IL-6 production, and (**C**) IL-10 production in the basolateral side was measured by ELISA. The depicted error bars represent the SD of 3 experiments. Data were analyzed using a one-way ANOVA followed by Tukey’s post hoc test, with statistical significance set at *p* < 0.05 (values sharing no common letter differ significantly).

To assess whether the LP22A-3 could create an anti-inflammatory environment, we evaluated the immunomodulating effect of an LP22A-3 administration. We used the intestinal co-culture model, which were described previously [25], consisting of a Caco-2 monolayer and differentiated naïve THP-1 cells on the apical and basolateral sides, respectively. Administration of LP22A-3 from apical site on the cell monolayer of Caco-2 inhibited TNF-α and IL-6 productions from LPS-stimulated THP-1 cells and prevented to decrease in IL-10 secretion into basolateral site (Figure 1). Our result suggested that LP22A-3 might create an anti-inflammatory environment.

### 3.2. Inhibitory Effect of the Oral Administration of LP22A-3 on ACD Model Mice

**Figure 2:**
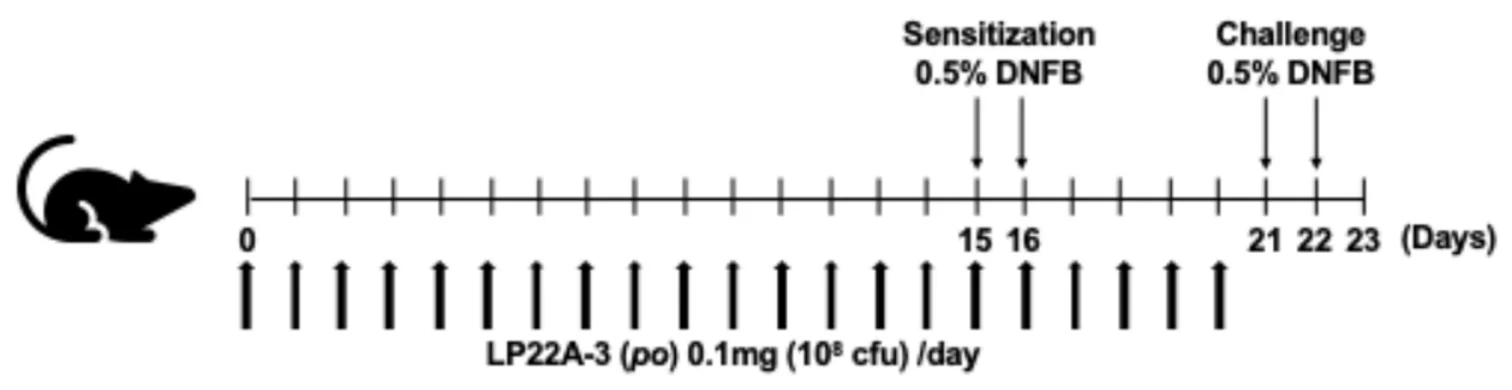
Experimental design for the oral LP22A-3 administration. Mice in the LP22A3 group were orally administered LP22A3 (1 × 10^8^ cfu/day) dissolved in 100 μL of 0.5% carboxymethyl cellulose sodium (CMC) solution every day until the day before challenging with DNFB (from Day 0 to 20). Mice were sensitized twice consecutively on the shaved abdominal sites with 50 μL of 0.5% DNFB (1-fluoro-2, 4-dinitrobenzene, Fuji Film Wako Pure Chemical Co., Ltd., Osaka, Japan) in acetone:olive oil (4:1) on Day 15 and 16. On Day 21 and 22 (six and seven days after the second sensitization), mice were consecutively challenged by painting the outer surfaces of both sides of each pinna with 20 μL of 0.5% DNFB. Vehicle groups received acetone:olive oil only. Each pinna was measured with a thickness gauge. The net pinna thickness was calculated at 24 h after the last challenge. Symptomatology of contact hypersensitivity was analyzed as ear thickness difference between at immediately prior to the first challenge and 24 h after the second challenge. All procedures were performed under anesthesia to prevent stress for the animals (1.5% isoflurane mixture oxygen).

**Figure 3:**
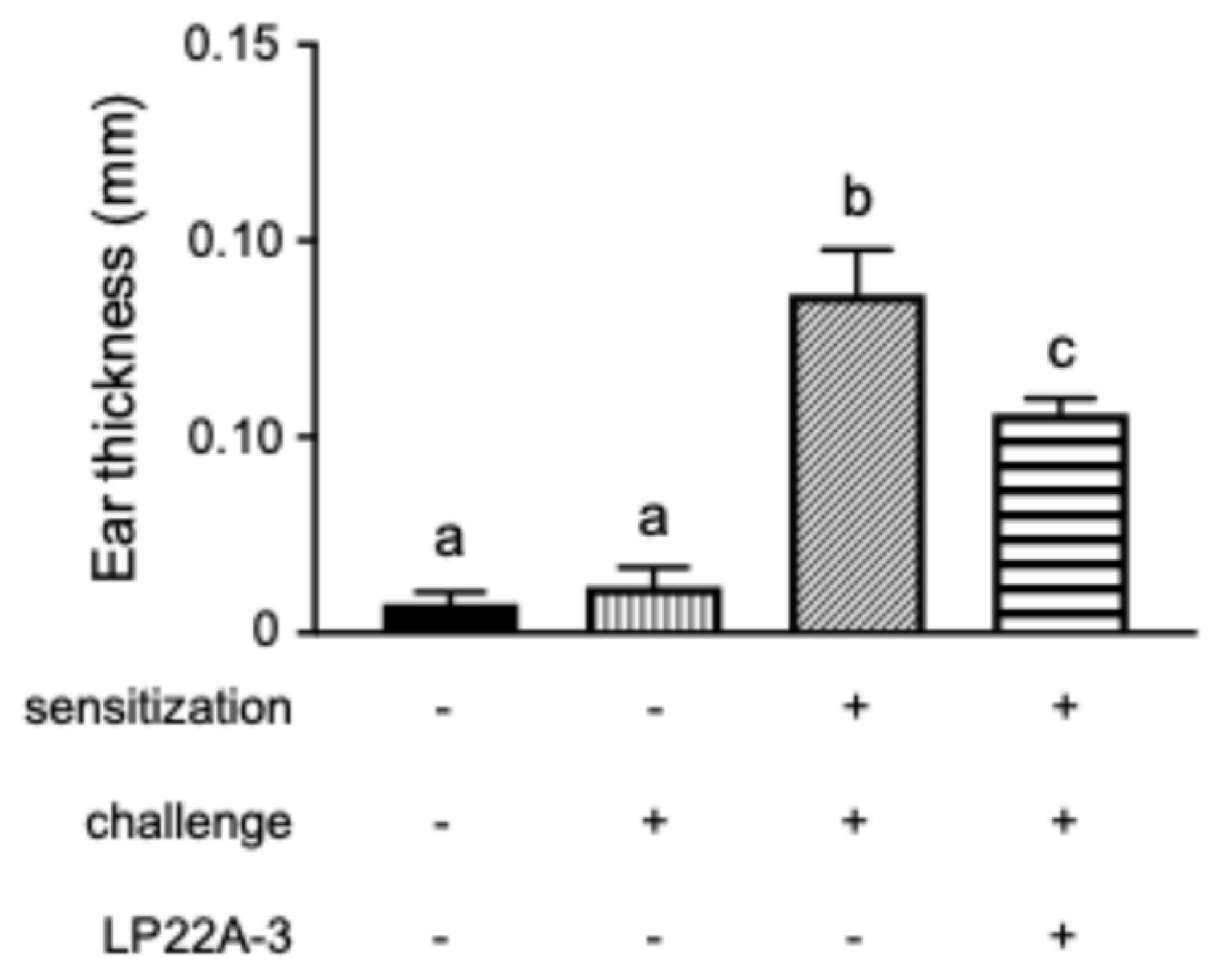
The oral LP22A-3 administration inhibits the edema caused by ACD progression. The mice were sensitized on two consecutive days (Day 15 and 16) with 0.5% DNFB (50 μL) on the shaved abdomen. After an incubation time of five days, the animals were initiated on two consecutive days (Day 21 and 22) on ears with 20 μL of 0.5% DNFB. The ear thickness was measured on the day immediately before the first challenge (Day 21) and 24 h after the second challenge. The difference between them was evaluated as the progression of edema. To assess the pathogenesis of ACD, the group without the sensitization by DNFB was employed. The depicted error bars represent the SEM (*n* = 5). Data were analyzed using a one-way ANOVA followed by Tukey’s post hoc test, with statistical significance set at *p* < 0.05 (values sharing no common letter differ significantly).

ACD is developed as a type IV allergic inflammation reaction. In vivo animal models were employed to delineate the systemic pathophysiological alterations associated with the onset of ACD and to elucidate the regulatory mechanisms exerted by the oral LP22A-3 administration (Figure 2). C57BL mice were sensitized with DNFB on the abdominal skin and challenged on the pinnae five days later, and then the increment in thickness of the pinnae in the mice was measured. To assess the pathogenesis of ACD, the group without the sensitization by DNFB was employed. Figure 3 demonstrates that ear swelling was significantly greater in mice challenged with DNFB than in the control mice. The oral administration of LP22A-3 suppressed ear edema caused by the challenge of DNFB. Therefore, the result suggests that LP22A-3 could exert an inhibitory effect on ACD as a functional food factor.

### 3.3. Effect of Oral LP22A-3 Administration on Cell Population in the Peritoneal Cavity of Mice

**Figure 4:**
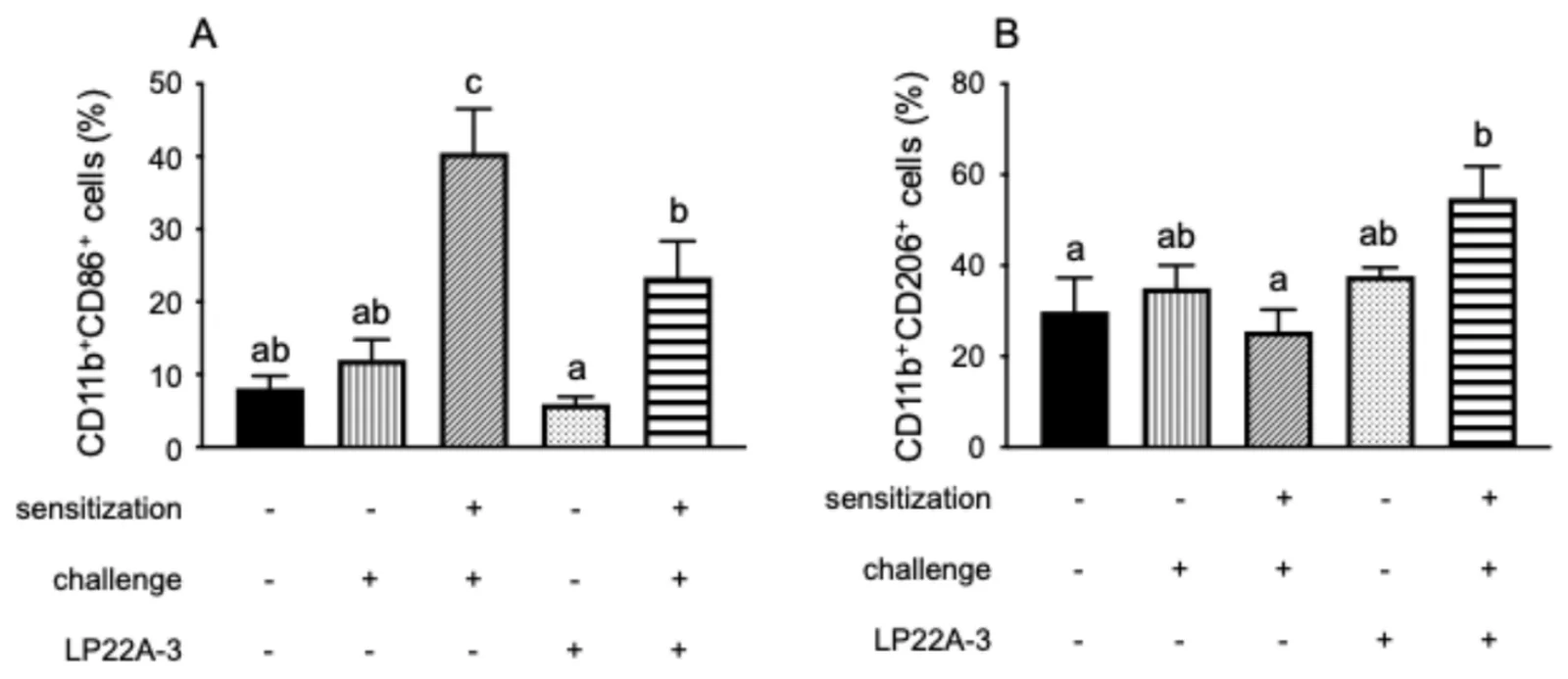
LP22A-3 regulates the differentiate of immunocompetent cells in the peritoneal cavity of the mice with DNFB-induced ACD. We analyzed the profiles of differentiated cells in the peritoneal cavity of the mice. The mice were sensitized on two consecutive days (Day 15 and 16) with 0.5% DNFB (50 μL) on the shaved abdomen. After an incubation time of five days, the animals were initiated on two consecutive days (Day 21 and 22) on ears with 20 μL of 0.5% DNFB. The mice were sacrificed on Day 23, then the cells were collected by peritoneal lavage with 10 mL of serum-free RPMI 1640 medium. The cells were washed once with fresh medium and were filtered using a 40 μm filter to obtain a single-cell suspension. Every cell was examined by a flow cytometry using APC-conjugated anti-CD11b, APC/Cy7-conjugsted anti-CD86, and PE/Cy7-conjugated anti-CD206 mAbs. (**A**) The differentiation toward CD11b^+^CD86^+^ cells induced by ACD onset was significantly attenuated, whereas (**B**) the differentiation toward CD11b^+^CD206^+^ cells was increased in the peritoneal cavity. To assess the influence of the pathogenesis of ACD, the group without the sensitization by DNFB was employed. The depicted error bars represent the SEM (*n* = 5). Data were analyzed using a one-way ANOVA followed by Tukey’s post hoc test, with statistical significance set at *p* < 0.05 (values sharing no common letter differ significantly).

To quantify the effect of LP22A-3 on differentiation of immunocompetent cells by oral administration, we analyzed the cell profiles in the peritoneal cavity of the mice. To assess the influence of the pathogenesis of ACD, the group without the sensitization by DNFB was employed. We found that the CD11b^+^CD86^+^ cells were significantly enhanced (Figure S1) by DNFB challenge (Figure 4A). On the other hand, it seemed that the CD11b^+^CD206^+^ cells (Figure S2) was slightly attenuated (Figure 4B). Those populations were completely reversed by oral administration of LP22A-3. As shown in Figure 4, the increase in CD11b^+^CD86^+^ cells induced by ACD onset was significantly attenuated by the LP22A-3 intake treatment, whereas CD11b^+^CD206^+^ cells were increased in the peritoneal cavity. These results suggest that the alleviative effect of LP22A-3 on edema forming could be attributed to the regulation of immunocompetent cell population in the mice with DNFB-induced ACD.

### 3.4. Effect of Oral LP22A-3 Administration on Treg Cells in the Spleen of Mice with DNFB-Induced ACD

**Figure 5:**
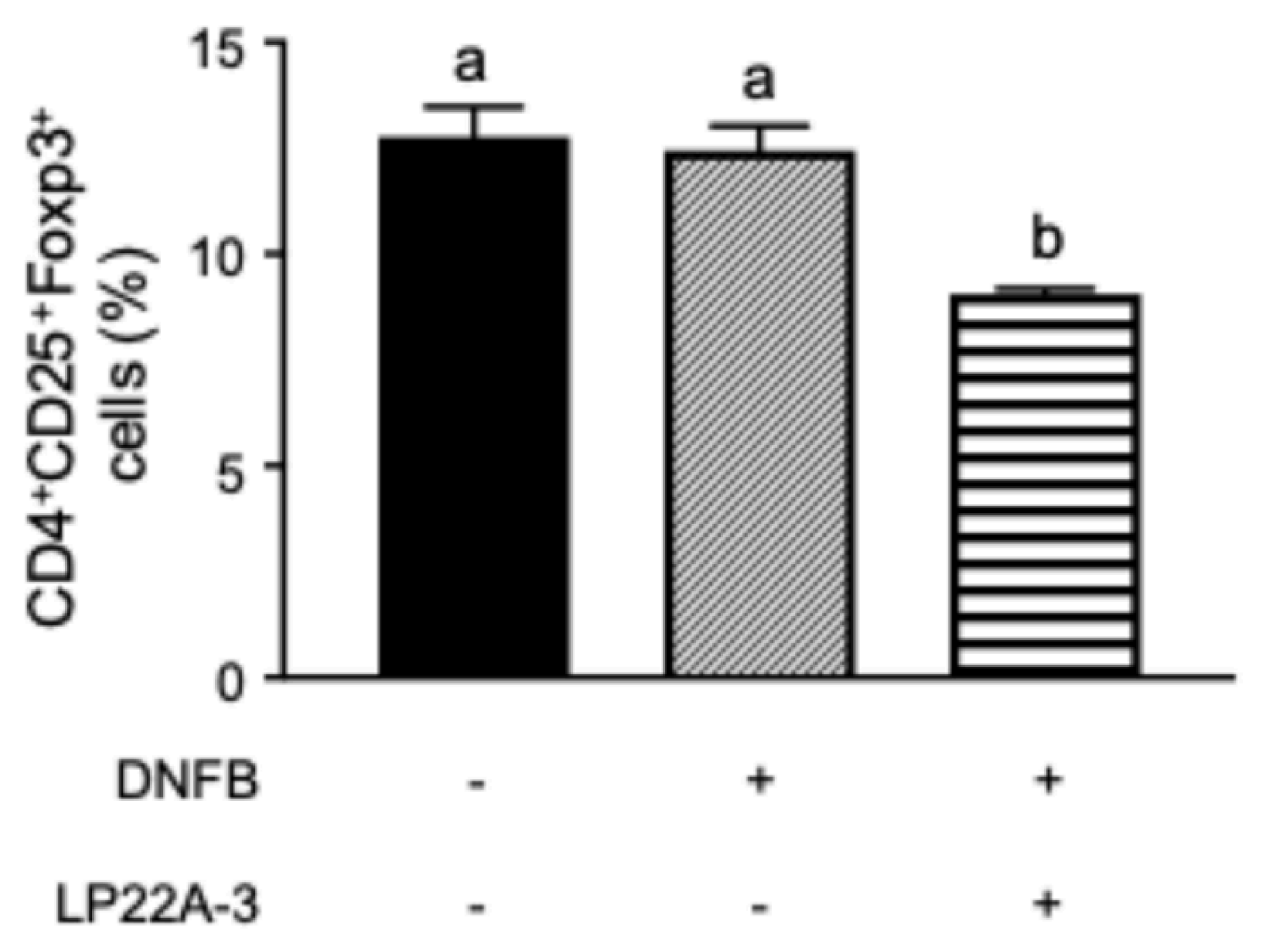
The oral LP22A-3 administration could affect the population of Treg cells in the spleen of the mice with DNFB-induced ACD. C57BL mice were sacrificed on Day 23 (24 h after the last challenge), and the spleen was extracted aseptically. Single-cell suspensions were obtained by disrupting the tissues between dissecting forceps in DMEM medium. After centrifuging, cells were cleared of erythrocytes by suspension on hypotonic lysis buffer for 30 min on ice. Cells were washed once with fresh medium and were filtered using a 40 μm filter to obtain a mononuclear suspension. Every cell was examined by a flow cytometry using FITC (for CD4)- and PE (for CD25)-mAbs. For intracelluer staining, the cells were fixed and permeabilized by using mouse permeabilized buffer set (BD Pharmingen) and then stained with APC-conjugated anti-Foxp3 antibodies. The depicted error bars represent the SEM (*n* = 5). Data were analyzed using a one-way ANOVA followed by Tukey’s post hoc test, with statistical significance set at *p* < 0.05 (values sharing no common letter differ significantly).

It has been reported that Treg cells play a crucial role in preventing inflammatory diseases such as colitis [25]. We have also indicated that the oral LP22A-3 administration elevated a proliferation of CD4^+^CD25^+^Foxp3^+^ cells (Treg cells) in the ileum of the mice with a gut inflammatory disease [21]. To examine the effect of oral LP22A-3 administration on systemic Treg cells, we evaluated the number of Treg cells in the spleen of mice with DNFB-induced ACD. We expected that the number could be systemically enhanced by a LP22A-3 intake. Unexpectedly, in this study, the oral administration of LP22A-3 decreased the number of CD4^+^CD25^+^Foxp3^+^ cells in the spleen, as shown in Figure 5. Further studies might be needed to evaluate how the change in Treg population was involved in alleviating the inflammation.

### 3.5. Effect of Oral LP22A-3 Administration on the Differentiation into the IL-9 Producing Cell from Naïve CD4^+^ T Cells In Vitro

**Figure 6:**
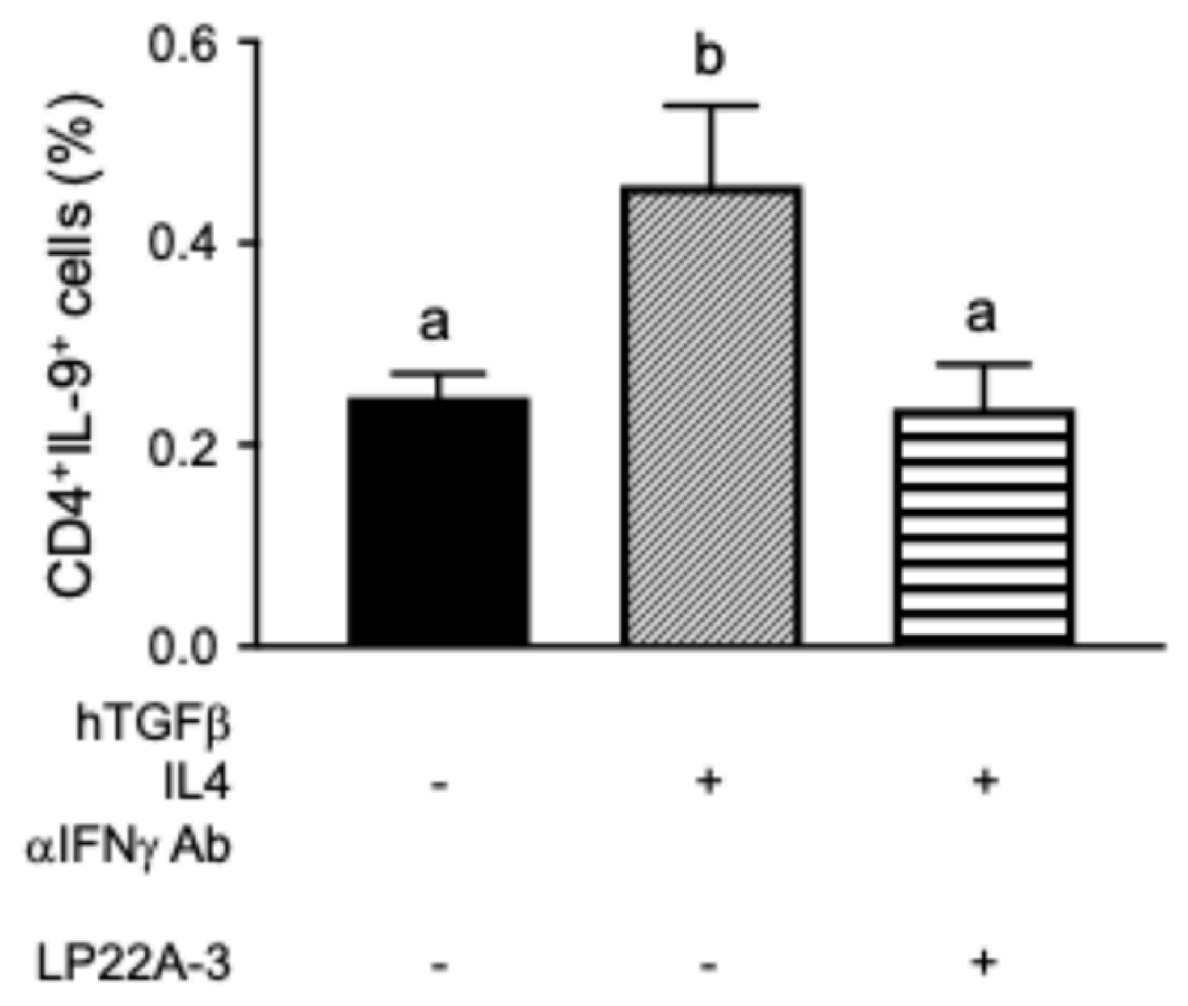
The oral LP22A-3 administration inhibits a differentiation of CD4^+^ IL-9-producing cells. The prepared naïve CD4^+^ T cells from the C57BL mice were stimulated with 3 μg/mL of anti-CD3 and 5 μg/mL soluble anti-CD28 and then polarized into Th9 cells under the condition with 3 ng/mL of hTGFβ, 20 ng/mL of IL-4, and 20 μg/mL of anti-IFNγ Ab [27]. Every cell was examined by a flow cytometry using FITC (for CD4)-mAb. For intracelluer staining, the cells were fixed and permeabilized by using mouse permeabilized buffer set (BD Pharmingen) and then stained with APC-conjugated anti-IL-9 antibodies. The depicted error bars represent the SEM (*n* = 5). Data were analyzed using a one-way ANOVA followed by Tukey’s post hoc test, with statistical significance set at *p* < 0.05 (values sharing no common letter differ significantly).

Previously, IL-9-producing CD4^+^ T cells might participate in immune-mediated diseases such as allergy and inflammatory diseases [30]. In this study, prepared naïve CD4^+^ cells from the spleen of C57BL mice administered orally with LP22A-3 were stimulated with anti-CD3 and soluble anti-CD28, and then polarized into IL-9-producing cells under the condition with hTGFβ, IL-4, and anti-IFNγ. As shown in Figure 6, the naïve CD4^+^ cells from non-LP22A-3-administered mice were induced to differentiate into IL-9-producing cells (Figure S3). The potential of differentiation was completely canceled by oral LP22A-3 administration. This result suggested that oral LP22A-3 administration inhibit to differentiate CD4^+^ cells toward IL-9-inducing cells; therefore, it might alleviate the inflammatory response in the DNFB-sensitized mice.

### 3.6. The Trafficking of the Cells from the Ileum Following Oral LP22A-3 Administration in Mice

**Figure 7:**
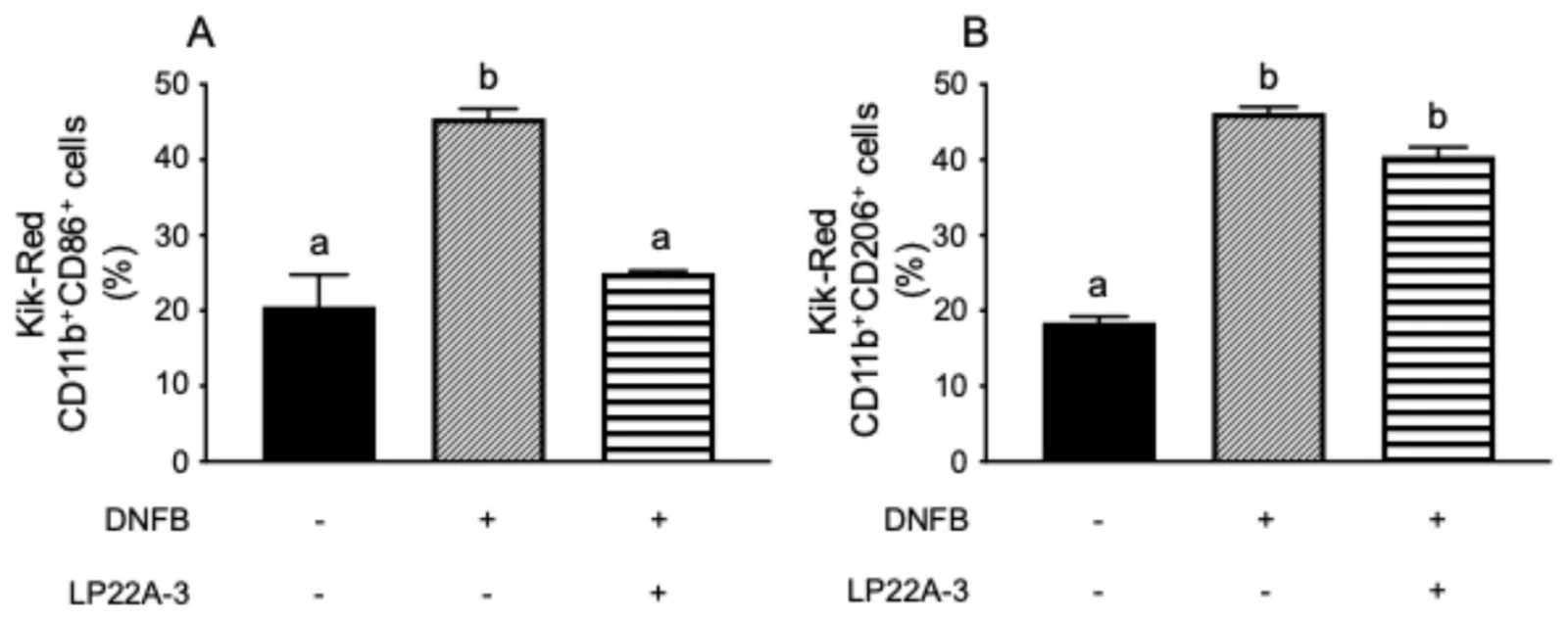
The oral administration of LP22A-3 regulates the migration of CD11b^+^CD86^+^/CD11b^+^CD206^+^ cells from the ileum of mice with DNFB-induced ACD. In this study, to quantify the effect of oral LP22A-3 administration on recruiting the cells in the small intestine of DNFB-induced ACD KikGR mice. The profiles of KikGR-red cells were evaluated in the axillary lymph nodes which was located adjacent to the pinnae. The ileum of the KikGR mice was exposed to violet light (405 nm, 54 mW/cm^2^) to induce photoconversion of the KikGR protein from green to red (KikGR-Red). (**A**) The oral LP22A-3 administration could ameliorate the accumulation of the red CD11b^+^CD86^+^ cells. On the other hand, (**B**) the CD11b^+^CD206^+^ cells increased in the ACD mice as well as CD11b^+^CD86^+^ cells did. The depicted error bars represent the SEM (*n* = 5). Data were analyzed using a one-way ANOVA followed by Tukey’s post hoc test, with statistical significance set at *p* < 0.05 (values sharing no common letter differ significantly).

To quantify the effect of oral LP22A-3 administration on the recruitment of the immune cells in the small intestine of DNFB-induced ACD mice, we analyzed the profiles of KikGR-red cells, which migrated from the ileum, in the axillary lymph nodes located adjacent to the pinnae (Figure 7). Exposure of the ileum in KikGR mice to 405 nm violet light triggered the photoconversion of the KikGR protein from green to red (KikGR-Red, Figure S4). As shown in Figure 7A and Figure S5, we found that the red CD11b^+^CD86^+^ cells (migration of the cells, Figure S5) from the ileum increased in the DNFB-induced ACD mice. Then, the oral LP22A-3 administration could reduce to baseline levels of the red CD11b^+^CD86^+^ cells from the ileum. On the other hand, the migration of red CD11b^+^CD206^+^ cells (the accumulation of KikGR-Red cells, Figure S6) enhanced in the ACD mice as well as the red CD11b^+^CD206^+^ cells did. However, it was shown that the increase in migration of those cells was not influenced by the LP22A-3 administration (Figure 7B). These results suggested that the immunocompetent cell migration might be fluctuated by the oral administration of LP22A-3 in the mice during alleviating DNFB-induced ACD.

## 4. Discussion

In the current study, we demonstrated that LP22A-3 exerts an inhibitory effect on allergic cutaneous inflammation by promoting the polarization of macrophages toward an anti-inflammatory phenotype. In an in vitro co-culture model consisting of THP-1 and Caco-2 cells, LP22A-3 significantly suppressed TNF-α and IL-6 secretion, whereas it promoted IL-10 production from LPS-stimulated matured THP-1 cells (Figure 1). We have previously reported that the LP22A-3 administration regulates productions of several cytokines from macrophages through stimulating intestinal epithelial cells in an in vitro experiment with co-culture system [20,21]. Moreover, it has indicated that the monocytes were induced to differentiate toward anti-inflammatory type of macrophages (M2 macrophages) under condition which anti-inflammatory cytokines were accumulated [31]. These findings suggested that LP22A-3 could induce to differentiate monocytes toward M2 macrophages through building anti-inflammatory condition. Intercellular adhesion between adjacent intestinal epithelial cells is mediated by tight junction complexes. Therefore, in the current study, we speculate that LP22A-3 must act on THP-1 cells though the Caco-2 monolayer rather than act directly. We have previously demonstrated that LP22A-3 interacted with Caco-2 cells through stimulating TLR2 on the surface, then TGF-β1 production was promoted from Caco-2 cells. It has been suggested that the TGF-β1 secretion by LP22A-3 administration was involved in IL-10 production in the mice with an inflammatory disease [21]. Although it is possible that the upregulation of TGF-β1 in intestinal epithelial cells leads to an anti-inflammatory condition, further study is required to elucidate the effect of TGF-β1 on macrophage differentiation.

Our previous study demonstrated that the oral LP22A-3 administration was a promising alleviative strategy for a colitis [21]. In the current study, we have shown that the oral administration of LP22A-3 possessed a possibility of preventing ACD. It is known as type IV allergy (delayed type hypersensitivity reaction) caused by exposure to exogeneous substances such as UV, fragrance, jewelry, clothes etc. The development of ACD proceeds through a biphasic mechanism consisting of an initial sensitization phase and a subsequent elicitation phase. During sensitization, the immune system undergoes primary priming to the allergen, which subsequently dictates the elicitation phase upon secondary allergen encounter. In the sensitization phase, several innate immune cells, including Langerhans cells and dendritic cells, are involved in ACD pathogenesis. On the other hand, macrophages, mast cells, innate lymphoid cells, neutrophils, eosinophils, and basophils elicit a delayed hypersensitivity reaction. Especially, macrophages have received a lot of attention as key cell types that contribute to a modulation of ACD pathogenesis. They are classified into two types: M1 macrophages classically activate an inflammatory condition and M2 macrophages are known to possess an anti-inflammatory property [13]. Furthermore, M2 macrophages are subclassified into four types, M2a, M2b, M2c, and M2d. It has been suggested that M1 macrophages are involved in a pathogenesis of ACD by producing inflammatory cytokines, whereas M2b type could contribute to prevent ACD exacerbation through secreting IL-10 [3]. In this study, we found that the oral LP22A-3 administration inhibited an upregulation of CD11b^+^CD86^+^ cell-differentiation in mice with DNFB-induced ACD. On the other hand, the differentiation toward CD11b^+^CD206^+^ cells was promoted in the peritoneal cavity of the mice (Figure 4). These results suggested that the oral LP22A-3 administration biased monocytes toward CD11b^+^CD206^+^ cell-differentiation through progressing anti-inflammatory environment in the ACD-induced mice. That might be why the oral administration of LP22A-3 prevents an inflammatory response in the ACD mice. However, in order to verify this effect of cell differentiation on alleviating inflammation, the definition for differentiated macrophages is required. Moreover, further experiment with macrophage depletion is necessary.

Under inflammatory condition, monocytes are recruiting to the inflammatory site [9]. We hypothesized that the oral administration of LP22A-3 could stimulate a chemotaxis mechanism of innate immune cells, and those cells play a critical role in alleviating the development of dermatitis. Therefore, quantifying the endogenous cell migration is fundamental to understanding that alleviation mechanism for dermatitis. To evaluate the immune cell migration, KikGR mice were used in this study [28] and the transition of cells from the ileum was analyzed using FACS analyzer. We showed here that the oral LP22A-3 administration could prevent the migration of CD11b^+^CD86^+^ cells from the ileum during the development DNFB-induced ACD (Figure 7A). Simultaneously, the migration of CD11b^+^CD206^+^ cells did not increase (Figure 7B). It has been reported that immature monocytes are recruited to the site of inflammation and differentiated there into matured macrophages [9]. In addition, there is a possibility that tissue-resident macrophages act as an anti-inflammatory macrophage. Our results in this study suggest that the oral administration of LP22A-3 could regulate a differentiation of immunocompetent cells and their migration. However, further studies are needed to clarify whether the macrophages were differentiated into anti-inflammatory (M2) macrophages, and whether the LP22A-3 administration is involved in regulating their migration.

Our previous study indicated that the oral LP22A-3 administration elevated a proliferation of CD4^+^CD25^+^Foxp3^+^ cells (Treg cells) in the intestine of the mice with a gut inflammatory disease [21]. In addition, it has been reported that the Treg cells migrated into the inflammatory site from the ileum to prevent inflammation in the mice with colitis [25]. It has been reported that Foxp3^+^ Treg cells play a crucial role in regulating inflammation in the intestine [32]. Therefore, we expected that the action of Treg cells could be systemically enhanced by the oral administration of LP22A-3 during inflammation. However, in this study, the unexpected decrease in the number of Treg was shown in the spleen of the DNFB-sensitized ACD mice with the LP22A-3 intake, compared to that in non-administered mice (Figure 5). Further studies are required to elucidate how Treg population is involved in alleviating ACD.

Veldhoen et al. [30] had defined the T helper cells producing IL-9 as Th9 cells. Th9 cells are a distinct Th cell lineage and resemble traditional Th2 cells [33]. Nevertheless, it is suggested that those cells induce the expression of multiple pro-inflammatory cytokines, including IL-9, IL-17, and IFNγ. Proliferation of Th9 cells induces an internal environment toward the proinflammatory mode and elicits pathogenesis of inflammatory diseases. Previous studies pointed to a positive correlation between a proliferation of Th9 cells and an exacerbation of inflammation [27]. In addition, Th9 cells are known to be skin-resident and produce TNF-α. Therefore, Th9 cells could be associated with pathogenesis of inflammation in the skin. Additionally, Th9 cells influence the progression of various inflammatory diseases. These findings establish the therapeutic value of targeting Th9 cells, providing novel approaches for precision treatment of inflammation. We here indicated that the oral LP22A-3 administration could inhibit a progression of CD4^+^ IL-9-producing cells differentiation in the DNFB-induced ACD mice (Figure 6). However, to sufficiently verify Th9, further experiments are required, such as the exclusion of other Th cells, Th1, Th2, Th17 and Treg, etc., and the measurement of specific transcription factor like PU.1 [27].

Our recent study has suggested that LP22A-3 initiated the protective effect on the ileum and ameliorated inflammatory diseases [21]. It also suggested that LP22A-3 affected anti-inflammatory potential of various immune cells in the lamina propria (LP) of the small intestine through stimulating TLR2 which predominantly expresses on the epithelial cells of the small intestine. In addition, it has been revealed that the migration of immunocompetent cells, such as T cells, was mediated by a G protein-linked chemoattractant receptor, GPR15, which is upregulated by TGF-β [25]. We have already shown that LP22A-3 intake increased the level of TGF-β1 in the mice with an inflammatory disorder [20,21]. Therefore, the oral administration of LP22A-3 might influence the cell migration from a small intestine by creating TGF-β1-rich conditions. The current study suggested that the oral LP22A-3 administration modulated the properties of the immune cells in the small intestine during the inflammation. We demonstrated that the oral LP22A-3 administration was a promising alleviative strategy for ACD and possibly other type-IV allergic disorders. Further studies are needed to clarify the mechanism of macrophage migration and the involvement of T lymphocytes in the pathogenesis of inflammatory diseases.

## 5. Conclusions

The oral administration of *Lactiplantibacillus plantarum* 22A-3 (LP22A-3) could suppress ear inflammatory edema in the mice sensitized with an allergy-induced chemical compound, DNFB. In this study, we found the migration of CD11b^+^CD86^+^ cells from the ileum of DNFB-induced ACD mice. The oral LP22A-3 administration could ameliorate the accumulation of CD11b^+^CD86^+^ cells. On the other hand, the migration of CD11b^+^CD206^+^ cells enhanced in the ACD mice. These results suggested that the DNFB-induced ACD might be attenuated through regulating of the migration of CD11b^+^CD86^+^ and CD11b^+^CD206^+^ cells by the oral administration of LP22A-3. There are too many unknown points regarding immune cell migration from the small intestine. Therefore, we aimed to clarify the mechanism in more detail by conducting experiments using KikGR mice, which allow us to track the migration of target cells. However, regarding the population of anti/pro-inflammatory macrophage, further verification is necessary. In addition, the oral LP22A-3 administration could be a promising alternative alleviation strategy for ACD and possibly other type-IV allergic disorders.

## 6. Limitation

We showed the immune cell populations by detecting CD11b^+^CD86^+^ and CD11b^+^CD206^+^ cells. However, those cells could not be sufficiently verified in vivo. Therefore, regarding the differentiation of M1/M2 macrophage in vivo, further verification is necessary by examining their secretions, such as pro/anti-inflammatory cytokines. Moreover, in order to verify the effect of macrophage differentiation on alleviating inflammation, further experimentation with macrophage depletion is necessary. In addition, further studies might be needed to evaluate how the change in Treg population was involved in alleviating the inflammation. In this study, we also showed that the ileum-derived cells were detected in the axillary lymph nodes near the inflamed ear tissue in the KikGR experiment. However, they could not be verified to migrate into the inflammatory sites. Therefore, regarding the migration of anti/pro-inflammatory macrophage, further verification is necessary. Furthermore, our results suggested that oral LP22A-3 administration inhibits the differentiation of CD4^+^ cells into IL-9-inducing cells. However, this study could not sufficiently characterize CD4^+^IL-9^+^ cells as Th9 cells. To sufficiently verify Th9, further experiments are required, such as the exclusion of other Th cells, and the measurement of specific transcription factor.

## Data Availability

Data can be obtained from the corresponding authors, K.-i.M., upon reasonable request.

## Supplementary Materials

The following supporting information can be downloaded at: https://www.mdpi.com/article/10.3390/nu18203446/s1, Figure S1: (A) Representative flow cytometric plots gated on monocyte, (B) Frequency of CD11b^+^ and CD86^+^ cells gated on monocyte; Figure S2: Representative flow cytometric plots gated on monocyte. Frequency of CD11b^+^ and CD206^+^ cells gated on monocyte; Figure S3: Representative flow cytometric plots gated on CD4^+^ lymphocyte. Frequency of IL-9 and CD4^+^ cells gated on CD4^+^ cells; Figure S4: Expression of KikGR-Green and KikGR-Red in the murine monocytes before and after photoconversion. Upon exposure violet light for 2 min, cells were converted from green (KikGR-Green) to red (KikGR-Red); Figure S5: Representative flow cytometric plots gated on KikGR-Red monocyte. Frequency of CD11b^+^ and CD86^+^ cells gated on KikGR-Red cells; Figure S6: Representative flow cytometric plots gated on KikGR-Red monocyte. Frequency of CD11b^+^ and CD206^+^ cells gated on KikGR-Red cells.

## Abbreviations

The following abbreviations are used in this manuscript:

ACD	allergic contact dermatitis
CD	contact dermatitis
cfu	colony forming unit
DNFB	1-fluoro-2, 4-dinitrobenzene
IL	interleukin
LP22A-3	*Lactiplantibacillus plantarum* 22A-3
TGF-β	transforming growth factor-β
TLR	toll-like receptor
TNF-α	tumor necrosis factor-α
