# Supplementary material for: The Alleviative Effect of Oral Administration of Lactiplantibacillus plantarum 22A-3 on Allergic Contact Dermatitis in Mice
Source: Nutrients. 2026 Oct 20;18(20):3446. doi: 10.3390/nu18203446 (PMC13708430; doi:10.3390/nu18203446)
Supplement: Supplementary file 1 [file nutrients-18-03446-s001.zip › nutrients-4553054-supplementary.pdf]

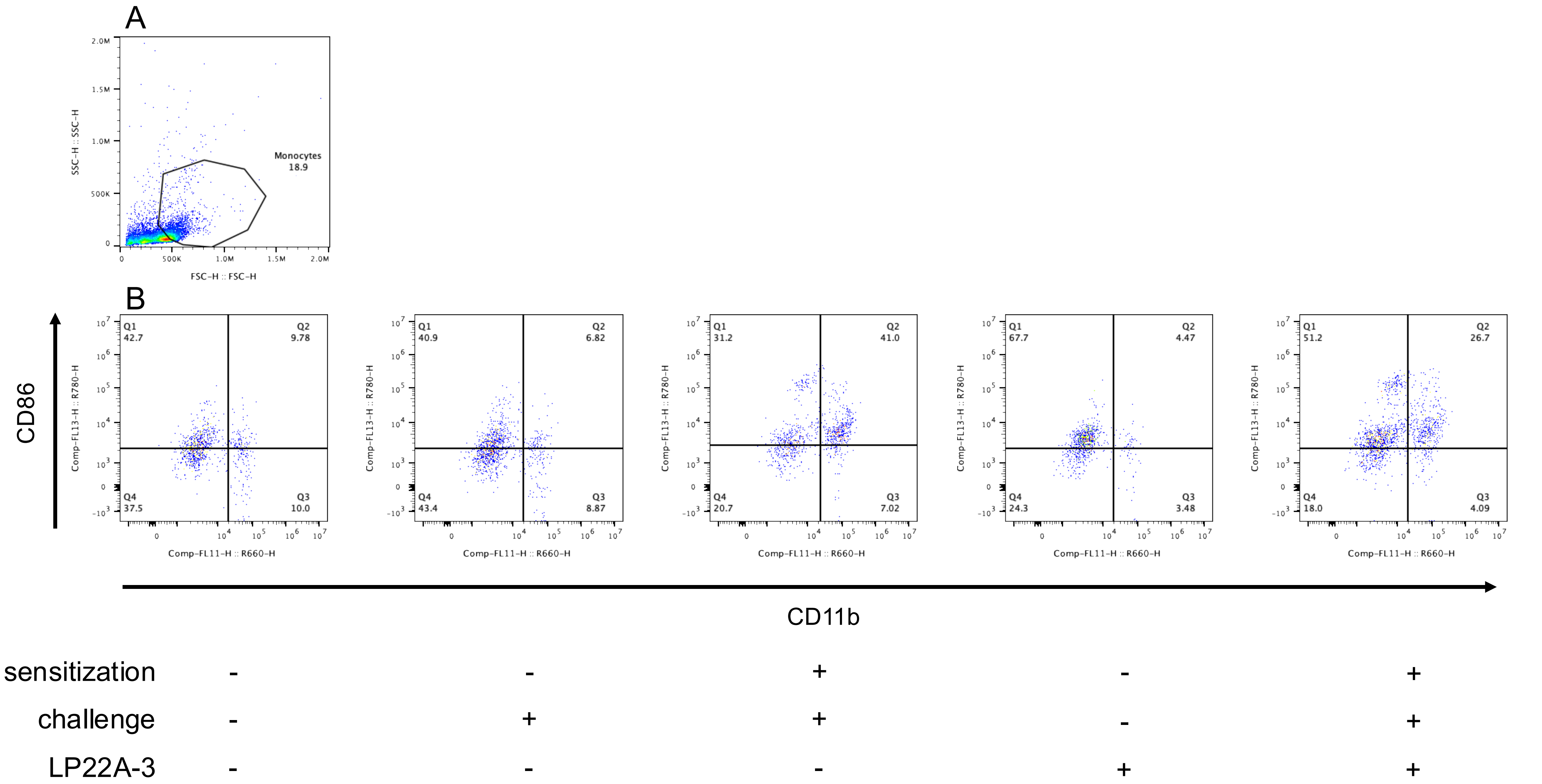

Figure S1. (A) Representative flow cytometric plots gated on monocyte, (B) Frequency of CD11b^{+} and CD86^{+} cells gated on monocyte

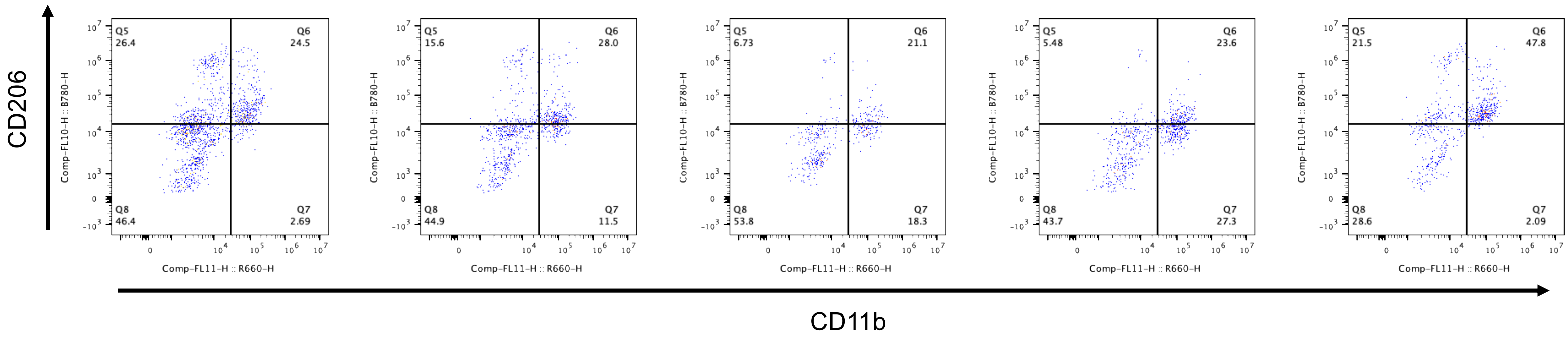

sensitization	-	-	+	-	+
challenge	-	+	+	-	+
LP22A-3	-	-	-	+	+

Figure S2. Representative flow cytometric plots gated on monocyte. Frequency of $CD11b^+$ and $CD206^+$ cells gated on monocyte

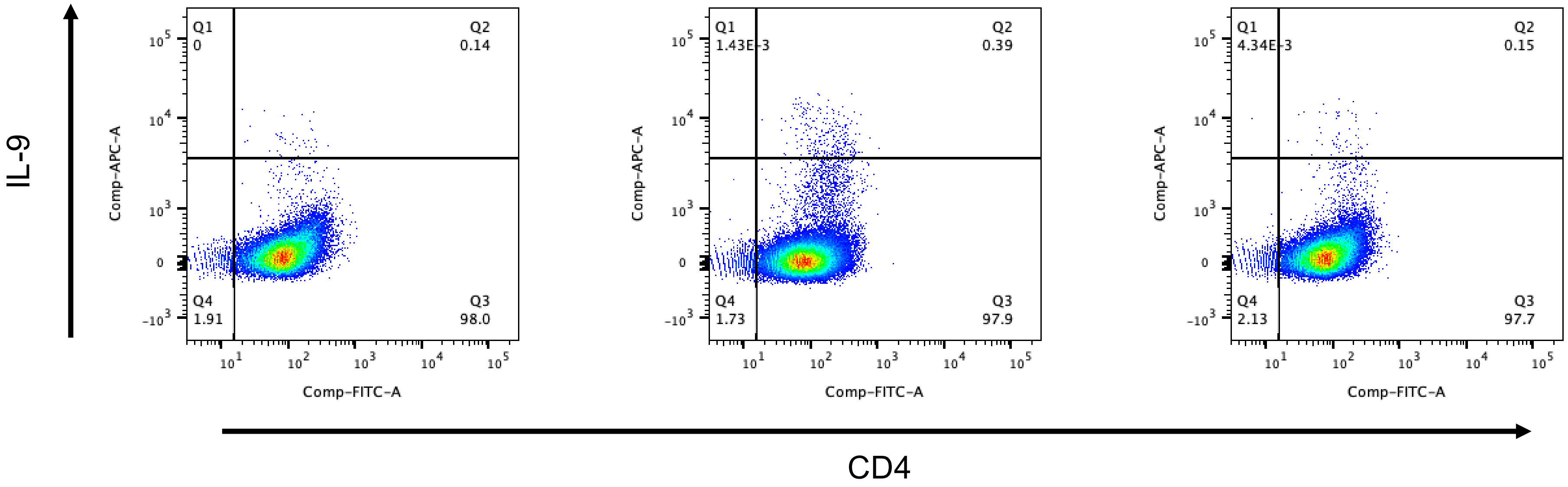

hTGFβ+IL4	-	-	+
αIFNγ Ab	-	+	+
LP22A-3	-	-	-

Figure S3. Representative flow cytometric plots gated on CD4^{+} lymphocyte. Frequency of IL-9 and CD4^{+} cells gated on CD4^{+} cells

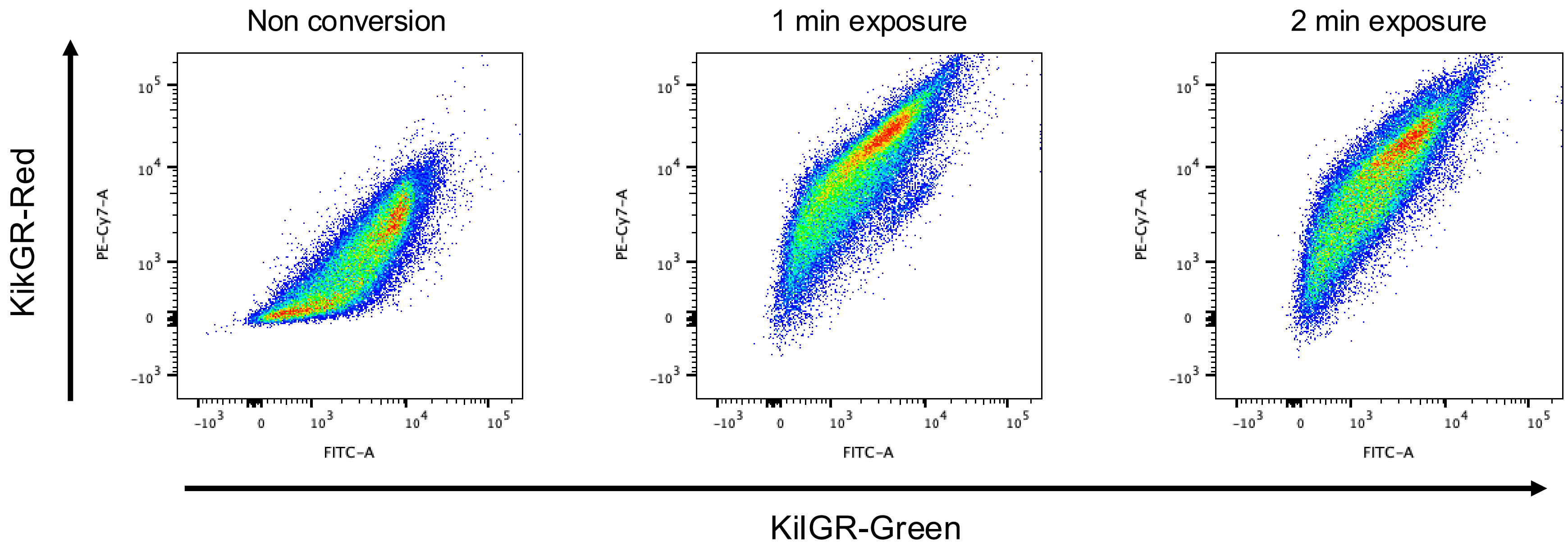

Figure S4. Expression of KikGR-Green and KikGR-Red in the murine monocytes before and after photoconversion. Upon exposure violet light for 2 minutes, cells were converted from green (KikGR-Green) to red (KikGR-Red).

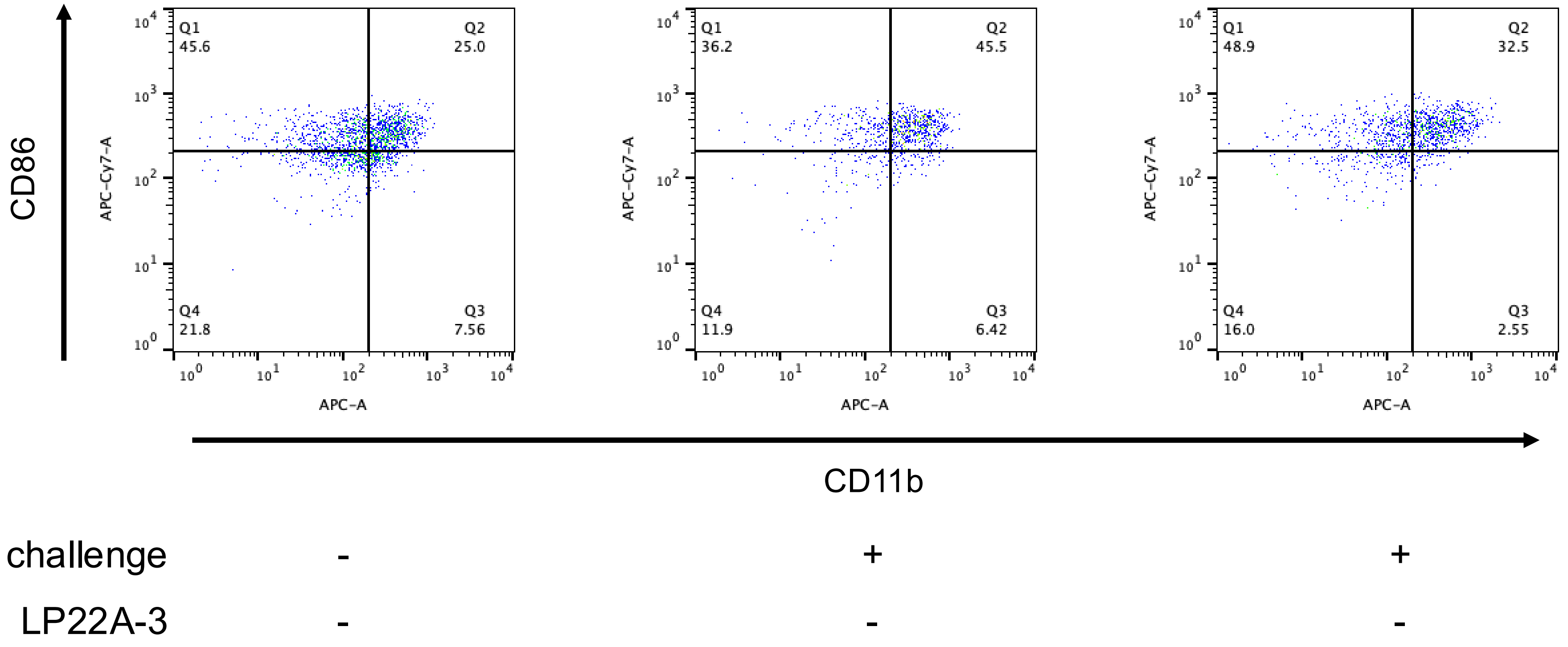

Figure S5. Representative flow cytometric plots gated on KikGR-Red monocyte. Frequency of CD11b^{+} and CD86^{+} cells gated on KikGR-Red cells

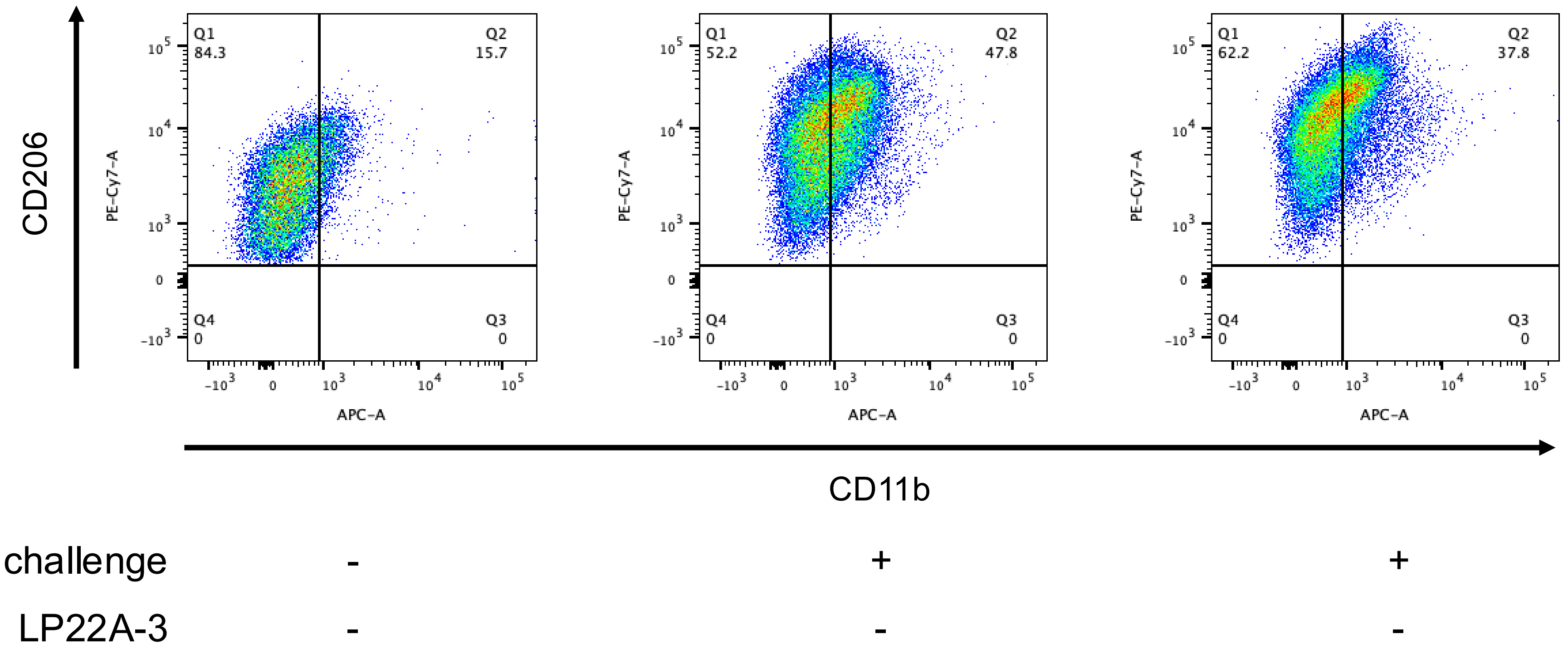

Figure S6. Representative flow cytometric plots gated on KikGR-Red monocyte. Frequency of $CD11b^{+}$ and $CD206^{+}$ cells gated on KikGR-Red cells
